# Contact tracing of syphilis-seropositive pregnant women and syphilis-infection among their male partners in Bao'an district, Shenzhen, China

**DOI:** 10.1186/s12879-020-05403-x

**Published:** 2020-09-18

**Authors:** Ruilin Yan, Baoqing Deng, Guichun Wen, Licheng Huang, Limei Li, Zhiming Huang

**Affiliations:** Shenzhen Bao’an Center for Chronic Disease Control, Shenzhen, China

**Keywords:** Syphilis, Pregnancy, Contact tracing, Partner notification

## Abstract

**Background:**

Untreated male partners are a critical source of maternal re-infection. Contact tracing is a good way to identify infection among partners and reduce risk of mother-to-child transmission related to maternal re-infection. This study aimed to analyze the current situation and related factors of contact tracing of syphilis-seropositive pregnant women and syphilis-infection among their male partners.

**Method:**

Data of syphilis-seropositive pregnant women and their male partners attending clinic for syphilis-screening were obtained from the Shenzhen Program for Prevention of Congenital Syphilis. Contact tracing rate of syphilis-seropositive pregnant women and syphilis prevalence among male partners were counted, and related factors were also analyzed using a random-effects logistic regression model.

**Result:**

Of the 1299 syphilis-seropositive pregnant women, 74.1% (963/1299) had their male partners receiving syphilis-screening and 19.1% (184/963) of male partners were syphilis-infected. For pregnant women, being divorced (adjusted odds ratio [AOR] =0.39; 95%CI: 0.17–0.87), seeking for emergency services at their first antenatal clinics visits (AOR = 0.58; 95%CI: 0.44–0.77), reporting willingness to notify partner(AOR = 7.65; 95%CI: 4.69–12.49), multi-partners (AOR = 1.38; 95%CI:1.03–1.86) and having a history of drug abuse (AOR = 0.37; 95%CI: 0.14–1.00)were independently associated with successful contact tracing. For male partners, of minority ethnicity (AOR = 4.15; 95%CI: 1.66–10.34), age at first sex>20(AOR = 0.57; 95%CI: 0.37–0.87), reporting multi-partners (AOR = 1.60; 95%CI: 1.04–2.46), having a history of drug abuse (AOR = 4.07; 95%CI: 1.31–12.64) were independently associated with syphilis-infection. In addition, pregnant women with TRUST titer ≥1:8 (AOR = 2.81; 95%CI: 1.87–4.21), having a history of adverse pregnancy outcomes (AOR = 1.70; 95%CI: 1.14–2.53), reporting multi-partners (AOR = 0.43; 95%CI: 0.29–0.64) and reporting the current partner as the source of syphilis (AOR = 5.05; 95%CI: 2.82–9.03) were independently associated with partners’ syphilis-infection.

**Conclusion:**

Contact tracing is feasible and effective in identifying syphilis-infected partners among syphilis-seropositive pregnant women. Contact tracing is associated with many factors such as women’s marital status, services at their first antenatal clinics visit and willingness of partner notification. Partners’ ethnicity, age at first sex, multi-partners and history of drug abuse as well as women’s levels of TRUST titer were associated with partners’ syphilis-infection.

## Background

Despite advances in syphilis prevention of mother-to-child transmission (PMTCT) [1, 2], pregnant women face a high risk of syphilis re-infection from infected male partners and adverse pregnancy outcomes (APOs) related to mother-to-child transmission [3–5]. It was estimated that globally there were 355,000 APOs caused by mother-to-child transmission of syphilis in 2016, of which 6% were due to treatment failures or re-infection during pregnancy [2]. Many male partners of syphilis-seropositive pregnant women are also syphilis-infected and do not know their syphilis status [3, 4]. Untreated male partners are a critical source of maternal re-infection, and pregnant women with syphilis-infected partners are more likely to have APOs [3, 5].

Contact tracing is the process of notifying index patients’ partners of possible exposure, and providing counseling, screening, diagnosis and treatment. As one of the most effective public health interventions in Sexually transmitted disease (STD) management, contact tracing was recommended by the Centers for Disease Control and Prevention (CDC) for all individuals presenting with STD [6]. In many countries, contact tracing was also included as an integral part of PMTCT program to identify partner infection, interrupt transmission, prevent re-infection, and reduce fetus mortality caused by syphilis and HIV infection [3, 4, 7].

In order to prevent mother-to-child transmission (MTCT) of syphilis, the Bureau of Health (now the Health Commission) of Shenzhen Municipality launched a program called the Shenzhen Program for Prevention of Congenital Syphilis (SPPCS) in 2001. All pregnant women are offered free syphilis-screening at their first antenatal clinic visits, and syphilis-seropositive pregnant women are encouraged to bring their male partners for further test and treatment [8]. Using data from the SPPCS, the present study described the current situation of contact tracing of syphilis-seropositive pregnant women and syphilis-infection among their male partners. We also explored factors associated with contact tracing of pregnant women and syphilis-infection among their male partners. Our findings may provide guidance for better contact tracing to identify infection among partners and reduce risk of MTCT related to maternal re-infection.

## Methods

### Recruitment of participants

The Shenzhen Program for Prevention of Congenital Syphilis (SPPCS) is a citywide program supported by the Bureau of Health (now the Health Commission) of Shenzhen Municipality. Initiated in 2001, SPPCS covers all antenatal clinics (ANCs) in Shenzhen city and offers free syphilis screening for all pregnant women at their first antenatal clinic visits. According to the SPPCS guidelines, syphilis-seropositive was defined as positive results in both toluidine red unheated serum test (TRUST, Shanghai Rongsheng Bio Tech Co, Ltd., Shanghai, China) and treponemal pallidum particle agglutination (TPPA, Fujirebio Inc., Tokyo, Japan [8]. All syphilis-seropositive pregnant women were encouraged to notify their male partners for further examinations. Three partner notifying approaches were recommended: patient referral, in which the pregnant women notify partners by themselves; provider referral, where doctors notify partners with pregnant women’s permission; contract referral, in which pregnant women agree to notify partners, but doctors may intervene if the partners do not present for treatment. Male partners presented to the clinics were recommended for syphilis-screening with both TRUST and TPPA, and if positive, were referred for further treatment [9]. Information of syphilis-seropositive pregnant women and male partner attending clinics for syphilis-screening were collected by the doctor using a self-developed structured questionnaire (presented in Additional files). The current study samples were drawn from SPPCS datasets and included pregnant women who were syphilis-seropositive and their male partners attending clinics for syphilis-screening in Bao’an District from 2012 to 2017. Considering the relatively stable sex relationships of pregnant women, their male partners traced in this study were limited to the closest one for each pregnant woman, mainly referring to spouse of the married and the closest male partner during the past 12 months for the unmarried or divorced. Syphilis-seropositive pregnant women and their male partners with complete information were finally included for analyses.

### Information collection

According to the SPPCS guidelines [8], syphilis-seropositive pregnant women were interviewed in a separate consulting room by the doctor and received a physical examination. Based on the results of the interview and physical examination, the doctor completed the self-developed structured questionnaire (presented in Additional files). Information of their male partners presenting to clinics for further examination was also collected by the doctor. Data of pregnant women and their male partners analyzed in the current study included: demographic characteristics (i.e., age, ethnicity, education, marital status, occupation, and residency status), history of past illness (i.e., history of drug abuse, history of syphilis or other STDs), obstetrical history (i.e., age at first sex, number of lifetime sexual partners, history of APOs), and history of present illness (i.e., pregnancy status at the first ANC visits, syphilis test results, staging diagnosis of syphilis, willingness to notify partner and partner notifying approaches).

### Statistical analysis

The primary outcomes of interest included contact tracing rate estimated as the proportion of syphilis-seropositive pregnant women whose partners received syphilis-screening, syphilis prevalence among male partners estimated as the proportion of male partners infected with syphilis, and their 95% confidence interval (CI). Characteristics of pregnant women listed in Table 1, as well as women’s staging diagnosis of syphilis, TRUST titer, willingness to notify partner and partner notifying approaches were tested as possible variables related to contact tracing. Characteristics of male partner listed in Table 1, as well as women’s staging diagnosis of syphilis, TRUST titer, number of lifetime partners and source of syphilis infection on pregnant women’s reports were tested as possible variables related to syphilis-infection among male partners. We explored potential variables related to contact tracing and syphilis-infection among male partners by using multivariate logistic regression models to estimate adjusted odds ratio (AOR) and 95% CIs. For all analyses, *P* values < 0.05 were regarded as statistically significant. All analyses were performed using SPSS for Windows software (version 17.0).

**Table 1:** Characteristics of syphilis-seropositive pregnant women and male partners

Characteristics	Pregnant women n (%)	Male partners n (%)
Age (Years)		
≤ 20	94 (7.2)	22 (2.3)
21–30	695 (53.5)	443 (46.0)
31–40	483(37.2)	403 (41.8)
> 40	27 (2.1)	95 (9.9)
Ethnicity		
Han	1252 (96.4)	939 (97.5)
Minority	47 (3.6)	24 (2.5)
Marital status		
Unmarried	203 (15.6)	–
Divorced	32 (2.5)	–
Married	1064 (81.9)	–
Occupation		
Employed	463 (35.6)	923 (95.8)
Unemployed	836 (64.4)	40 (4.2)
Education level		
Secondary school or below	895 (68.9)	489 (50.8)
Senior high school	301 (23.2)	326 (33.9)
College or above	103 (7.9)	148 (15.4)
Service at first ANC visit		
Routine antenatal care	936 (72.1)	–
Emergency services	363 (27.9)	–
Source of syphilis infection on pregnant women’s reports		
Current partners	379 (29.2)	–
Someone other than current partner	296 (22.8)	–
Unknown	624 (48.0)	–
Age at first sex (Years)		
≤ 20	418 (32.2)	429 (44.5)
>20	881 (67.8)	342 (35.5)
Unknown	0 (0.0)	192 (19.9)
Number of lifetime sex partners		
1	478 (36.8)	311 (32.3)
≥ 2	652 (50.2)	377 (39.1)
Unknown	169 (13.0)	275 (28.6)
History of APOs		
Yes	302 (23.2)	–
No	997 (76.8)	–
History of drug abuse		
Yes	20 (1.5)	18 (1.9)
No	1279 (98.5)	945 (98.1)
History of other STD		
Yes	14 (1.1)	8 (0.8)
No	1285 (98.9)	955 (99.2)

## Results

### Participant characteristics

Between January 1st, 2012 and December 31st^,^ 2017, 626,446 pregnant women were screened for syphilis and 1302 were identified to be syphilis-seropositive as having positive results in both TRUST and TPPA. A total of 1299 syphilis-seropositive pregnant women with complete available information were enrolled. 963 male partners of syphilis-seropositive pregnant women presented to clinic and received screening for syphilis. Data of 1299 syphilis-seropositive pregnant women and 963 male partners were included for analyses.

Characteristics of 1299 syphilis-seropositive pregnant women are presented in Table 1. The pregnant women had a mean age of 28.81 (SD = 5.85, range: 16–49). Most pregnant women presented to the clinics for routine antenatal care at their first ANC visits (*n* = 936, 72.1%), and the rest presented for emergency services, including in labor (*n* = 165, 12.7%), spontaneous abortion (*n* = 43, 3.3%), stillbirth (*n* = 25, 1.9%), ectopic pregnancies (*n* = 120, 9.2%), and artificial abortions or inductions of labor (*n* = 10, 0.8%). 10 (0.8%) women having existing symptoms of early syphilis in the physical examination at their first ANC visits, 419(32.3%) women having a history of syphilis and having been treated before pregnancy, and 870 (67.0%) women lacking existing symptoms of syphilis in the physical examination and without anti-syphilis treatment before pregnancy were diagnosed as early syphilis, syphilis after adequate treatment, and latent syphilis, respectively. TRUST titer ranged from 1:1–1:256, with the majority under 1:8 (*n* = 954, 73.4%). 418(32.2%) of pregnant women had first sex at the age of 20 and younger, and 652(50.2%) reported more than one lifetime sex partners.

Characteristics of 963 male partners are presented in Table 1. The male partners had a mean age of 31.50 (SD = 6.53, range: 17–56).429(44.5%) of male partners had first sex at the age of 20 or younger, and 377(39.1%) reported more than one lifetime sex partners.

### Contact tracing and associated factors

Among the 1299 syphilis-seropositive pregnant women, 1205(92.8%) stated willingness to notify their male partners, and 94 (7.2%) reported that they did not want to notify their male partners. The barriers to partner notify reported by the pregnant women included fear of relationship breakdown (*n* = 82, 87.2%), lack of contact information because of having divorced or separated with partners (*n* = 10, 10.6%), fear of social discrimination (n = 1, 1.1%), and distrust of the diagnosis (n = 1, 1.1%). 1191 (91.7%) of the syphilis-seropositive pregnant women selected patient referral, 66 (5.1%) selected provider referral and 42 (3.2%) selected contract referral. Finally, 1155(88.9%) pregnant women reported that their partners were notified, 985 (75.8%) partners presented to the clinics and 963 (74.1%) male partners received screening for syphilis, resulting in a contract tracing rate of 74.1% (95% CI, 72.9–75.3%).

Factors independently associated with contact tracing in a multivariate regression model are showed in Table 2. Pregnant women being divorced (adjusted odds ratio [AOR] =0.39; 95%CI: 0.17–0.87), seeking for emergency services at their first ANC visits (AOR =0.58; 95%CI: 0.44–0.77), reporting willingness to notify partner (AOR = 7.65; 95%CI: 4.69–12.49), multi-partners (AOR = 1.38; 95%CI: 1.03–1.86) and having a history of drug abuse (AOR = 0.37; 95%CI: 0.14–1.00) were independently and significantly associated with successful contact tracing.

**Table 2:** Multivariate logistic model for associated factors for contact tracing among syphilis-seropositive pregnant women

Characteristics	Pregnant women	Pregnant women whose male partner received syphilis screening n (%)	Adjusted OR(95%CI)	***P*** values
Marital status				
Married	1064	817 (76.8)	reference	–
Unmarried	203	131 (64.5)	0.83 (0.55–1.25)	0.374
Divorced	32	15 (46.9)	0.39 (0.17–0.87)	0.021
Service at first ANC visit				
Routine antenatal care	936	735 (78.5)	reference	–
Emergency services	363	228 (62.8)	0.58 (0.44–0.77)	<0.001
Willingness to notify partner				
No	94	30 (31.9)	reference	–
Yes	1205	933 (77.4)	7.65 (4.69–12.49)	<0.001
Number of lifetime sex partners				
1	478	345 (72.2)	reference	–
≥ 2	652	501 (76.8)	1.38 (1.03–1.86)	0.034
Unknown	169	117 (69.2)	1.01 (0.67–1.53)	0.969
History of drug abuse				
No	1279	953 (74.5)	reference	–
Yes	20	10 (50.0)	0.37 (0.14–1.00)	0.049

### Syphilis infection status among male partners and associated factors

Among the 963 male partners screened for syphilis, 779 (80.9%) were negative in both TRUST and TPPA, 155 (16.1%) were positive in both TRUST and TPPA, 29 (3.0%) were positive only in TPPA, leading to a syphilis prevalence of 19.1% (184/963).

Factors independently associated with syphilis infection among male partners in a multivariate regression model are shown in Table 3. For pregnant women, TRUST titer ≥1:8 (AOR = 2.81; 95%CI: 1.87–4.21), syphilis infection from the current partner (AOR = 5.05; 95%CI: 2.82–9.03), multi-partners (AOR = 0.43; 95%CI: 0.29–0.64), history of APOs (AOR = 1.70; 95%CI: 1.14–2.53) were all independently and significantly associated with syphilis-infection of their male partners. For the male partners themselves, of minority ethnicity (AOR = 4.15; 95%CI: 1.66–10.34), age at first sex > 20 years old (AOR = 0.57; 95%CI: 0.37–0.87), multi-partners (AOR = 1.60; 95%CI: 1.04–2.46), and history of drug abuse (AOR =4.07; 95%CI: 1.31–12.64) were all independently and significantly associated with their own syphilis-infection.

**Table 3:** Multivariate logistic model for associated factors for syphilis-infection among male partners

Characteristics	Male partners	Syphilis-infected n (%)	Adjusted OR (95%CI)	***P*** values
Pregnant women				
TRUST titer				
<1:8	712	115 (16.2)	reference	–
≥ 1:8	251	69 (27.5)	2.81 (1.87–4.21)	<0.001
Source of syphilis infection on pregnant women’s reports				
Someone other than the current partner	225	20 (8.9)	reference	–
Current partner	284	97 (34.2)	5.05 (2.82–9.03)	<0.001
Unknown	454	67 (14.8)	1.61 (0.91–2.88)	0.105
Number of lifetime partners				
1	345	91 (26.4)	reference	–
≥ 2	501	62 (12.4)	0.43 (0.29–0.64)	<0.001
Unknown	117	31 (26.5)	1.01 (0.59–1.73)	0.976
History of APOs				
No	728	125 (17.2)	reference	–
Yes	235	59 (25.1)	1.70 (1.14–2.53)	0.009
Partners				
Ethnicity				
Han	939	174 (18.5)	reference	–
Minority	24	10 (41.7)	4.15 (1.66–10.34)	0.002
Age at first sex (Years)				
≤ 20	429	93 (21.7)	reference	–
>20	342	51 (14.9)	0.57 (0.37–0.87)	0.009
Unknown	192	40 (20.8)	0.76 (0.46–1.23)	0.256
Number of lifetime sex partners				
1	311	52 (16.7)	reference	–
≥ 2	377	81 (21.5)	1.60 (1.04–2.46)	0.033
Unknown	275	51 (18.5)	1.26 (0.79–2.03)	0.336
History of drug abuse				
No	945	177 (18.7)	reference	–
Yes	18	7 (38.9)	4.07 (1.31–12.64)	0.015

## Discussion

Contact tracing was recommended by World Health Organization as a critical strategy of PMTCT [10]. In our study, 74.1% of male partners of syphilis-seropositive pregnant women received further screening for syphilis, similar to the contact tracing rate reported in Bolivia (76.9%) [11], and higher than that reported in Uganda (18%) [12], Peru (33.7%) [13], Botswana (52.9%) [4], Kenya (67%) [3] and that reported among non-pregnant women with STD (13.6–58%) [14–16]. 19.1% of the male partners screened for syphilis were syphilis-seropositive, of whom 60% were newly diagnosed, similar to the prevalence of 18.1% reported in Kenya [3]. The findings indicated the feasibility and efficacy of contact tracing for syphilis among pregnant women.

Consistent with previous studies, partners of pregnant women seeking for routine antenatal care were more likely to present to the clinics for further screening than partners of pregnant women seeking for emergency services due to parturiency, spontaneous abortion, stillbirth, ectopic pregnancy and artificial abortions or inductions. These findings indicate that couples are more compliant with recommendations of partner notification in consideration of their unborn child’s health [3, 13]. Pregnant women’s willingness to notify partners was associated with increased contact tracing. Moreover, it was reported in previous studies that poor understanding of disease was one of the barriers to contact tracing [12, 14], and contact tracing among pregnant syphilis patients increased from 29.9 to 88.8% after counseling [14]. These findings suggested that health workers should make it clear to the patients about the potential consequences of syphilis infection for their unborn child and emphasize the importance of partner screening and treatment in preventing APOs caused byre-infection. Compared to married women, divorced pregnant women were more likely to have a casual partner and often did not inform their partners or could not trace casual partners [3, 17].

Compared to those having first sex before 20 years old, partners having first sex after 20 usually had higher self-protection awareness, which decreased risk of syphilis-infection. Compared to Han, minority ethnicity (mainly Zhuang, Miao and Yao) were mostly rural-to-urban migration workers with increased risk of acquiring STIs due to insufficient reproductive health knowledge and inadequate health service utilization [18, 19]. Multi- partners and drug abuse were risk factors of acquiring STD. Drug abusers were involved in risk behaviors that may place them at increased risk of STD, such as promiscuity, multi-partners and unprotected sex. Moreover, intravenous drug use also increased risk of STD by blood-borne infection.

Historical data suggest that patients with high TRUST titers, as seen in earlier stages of infection, were at highest risk of passing on infections to their partners, and the risk of transmission decreased with the duration of infection [20, 21]. Women with TRUST titer ≥1:8 were highly infectious and more likely to transmit to their partners. On the other hand, as the infected women with TRUST titer ≥1:8 were more likely to be in earlier stages of infection, indicating new infection from the current relationship. Compared to pregnant women with multiple sex partners, women with only one sex partner were more likely to be infected from the current partners. For pregnant women with a history of APOs, partner screening should be valued and strengthened although the association between APOs and partners’ syphilis-infection have not been checked. In our study, 50.2% of pregnant women reported more than one lifetime sex partners and those sex partners may be an important source of infection. It is thus important to expand contact tracing by including prior sex partners and casual sex partners with exposure risks to identify more cases and further reduce re-infection.

Our study had several limitations. First, successful contact tracing depends on both partner notify and partner compliance. In the current study, we recruited male partners through pregnant women and analyzed only those presented to the clinics. Information of those who didn’t present to the clinics remains unknown. Second, male partners traced in our study were limited to spouse of married pregnant women and the closest male partners of the unmarried or divorced pregnant women. Future research may consider expanding partners by including all sex partners with exposure risks. However, the study provides implications for further interventions to improve pregnant women’s contact tracing and identify syphilis-infection in partners to reduce risk of mother-to-child transmission related to maternal re-infection.

## Conclusions

In this study, we found that 74.1% of male partners received further syphilis-screening and 19.1% of them were syphilis-seropositive, confirming feasibility and effectiveness of contact tracing for syphilis among pregnant women. Contact tracing was affected by many factors, including pregnant women’s marital status, services at their first ANC visits and willingness of partner notification. Partners’ syphilis infection was associated with pregnant women’s TRUST titer, partners’ ethnicity, drug abuse, age at first sex, as well as multi-partners. These findings provide implications for better contact tracing to identify infection among partners and reduce risk of mother-to-child transmission related to maternal re-infection.

## Supplementary information

**Additional file 1.** Self-developed structured questionnaire.

## Data Availability

The datasets used and/or analyzed during the current study are available from the corresponding author on reasonable request.

## Abbreviations

PMTCT: Prevention of Mother-To-Child Transmission
APOs: Adverse Pregnancy Outcomes
STD: Sexually transmitted disease
CDC: Centers for Disease Control and Prevention
MTCT: Mother-To-Child Transmission
SPPCS: Shenzhen Program for Prevention of Congenital Syphilis
ANCs: Antenatal Clinics
TRUST: Toluidine Red Unheated Serum Test
TPPA: Treponemal Pallidum Particle Agglutination
CI: Confidence Interval
OR: Odds ratio
AOR: Adjusted Odds Ratio
GW: Gestational Week
