# Supplementary material for: Contact tracing of syphilis-seropositive pregnant women and syphilis-infection among their male partners in Bao'an district, Shenzhen, China
Source: BMC Infect Dis. 2020 Sep 18;20:684. doi: 10.1186/s12879-020-05403-x (PMC7501691; doi:10.1186/s12879-020-05403-x)
Supplement: Supplementary file 1 — Additional file 1. Self-developed structured questionnaire. [file 12879_2020_5403_MOESM1_ESM.docx]

**Self-developed structured questionnaire**

**Information of pregnant women**

**Part 1 General information**

01 Name： ______

02 Age (years)： ______

03 Telephone number： ______

04 Ethnicity：______

05 Marital status：①Single②Married③Widowed④Divorced

06 Census register：①Shenzhen②Others

07 Occupations：①Homemaker or unemployed②Service staff③Self-employed④Laborer

⑤Driver⑥Company staff⑦Civil servant⑧Others_____

08 Education：①Primary school or below②Junior high school③Senior high school④College or above

**Part 2 History of past illness**

09 Have you ever taken drugs? ①Yes②No

10 Have you ever been diagnosed with syphilis?

①Yes, date of diagnosis _____

②No ( If "No", jump to question 11)

Have you ever received adequate anti-syphilis treatment？ ①Yes②No

11 Have you ever been diagnosed with other sexually transmitted disease？①Yes②No

**Part 3 Obstetrical history**

12 Age at first sex (Years):_____

13 Apart from your current sexual partner, have you had sex with anyone else? ①Yes②No

14 Have you ever had an adverse pregnancy outcome?

①No②Spontaneous abortion③Intrauterine fetal death④Preterm birth⑤Stillbirth⑥ Artificial abortion or induction of labor⑦Childbirth with congenital syphilis⑧Ectopic pregnancies

**Part 4 History of present illness**

15 Pregnancy status at the first ANC visits: ①During pregnancy②In labor③Spontaneous abortion④Intrauterine fetal death⑤Stillbirth⑥Ectopic pregnancies⑦Artificial abortion or induction of labor

16 Syphilis test results at the first ANC visit:

TRUST titer：_____ TPPA titer：_____

17 Existing symptoms of syphilis: ①Yes. Description of symptoms:_____②No

18 Staging diagnosis of syphilis: ①Primary syphilis②Secondary syphilis③Tertiary syphilis④Latent syphilis⑤Syphilis after adequate treatment

19 The most likely source of your syphilis infection in your opinion:

①Current partner②Someone other than current partner③Unknown

20 Would you like to inform your sexual partner about your syphilis infection?

①Yes②No. Description of the reason: ____

21 If it is necessary to inform your sexual partner, which of the following methods would you like to notify?

①Notify partner by myself ②Notify partner by doctor ③Notify partner by myself first, doctors can intervene if my sexual partner still has not been tested after being told

**Information of male sex partners**

(The sexual partner traced here refers to the male partner who has the closest relationship with the pregnant woman, mainly referring to the spouse of a married person and the sexual partner of an unmarried/divorced person who has the closest relationship in the last 12 months..)

22 Has the male sexual partner been tested for syphilis? ①Yes②No

23 Name： ______

24 Age (years)： ______

25 Telephone number： ______

26 Ethnicity：______

27 Census register：①Shenzhen②Others

28 Occupations：①Homemaker or unemployed②Service staff③Self-employed④Laborer⑤Driver⑥Company Staff⑦Civil servant⑧Others_____

29 Education：①Primary school or below②Junior high school③Senior high school④College or above

30 Age at first sex (Years)_____

31 Apart from your current sexual partner (pregnant woman), have you had sex with anyone else? ①Yes②No

32 Have you ever taken drugs? ①Yes②No

33 Have you ever been diagnosed with sexually transmitted disease other than syphilis?

①Yes②No

34 Results of this syphilis test:

TRUST：①Positive, titer_____② Negative

TPPA：①Positive，titer_____② Negative
